# A Narrative Review on Recombinant Human Bone Morphogenetic Protein 2: Where Are We Now?

**DOI:** 10.7759/cureus.67785

**Published:** 2024-08-26

**Authors:** John P Von Benecke, Elisa Tarsitano, Laura-Marie A Zimmermann, Kevin M Shakesheff, William R Walsh, Hyun W Bae

**Affiliations:** 1 Research, Locate Bio Limited, Nottingham, GBR; 2 Research, BioRizon Consulting Private Limited, Singapore, SGP; 3 School of Clinical Medicine, Prince of Wales Clinical School, University of New South Wales, Syndey, AUS; 4 Orthopaedics, Cedars-Sinai Medical Center, Los Angeles, USA

**Keywords:** spinal fusion, bone grafts, orthobiologics, side effects, trends, clinical usage, bone morphogenetic protein-2, rhbmp-2

## Abstract

Spinal fusion is a prevalent surgical intervention for degenerative spinal diseases, with increasing demand driven by ageing populations. The coexistence of multiple chronic conditions, termed multimorbidity, often complicates surgical outcomes, making advanced bone grafts crucial for successful fusions. This paper reviews the development, clinical application, and controversies surrounding the use of recombinant human bone morphogenetic protein-2 (rhBMP-2) in spinal fusion surgeries. A comprehensive narrative review was conducted, focusing on literature from January 1980 to January 2024, sourced from PubMed and Google Scholar. Studies included those examining rhBMP-2 specifically in spinal fusion contexts, excluding other bone morphogenetic proteins (BMPs) and non-spinal applications.

This review presents an overarching synopsis of rhBMP-2, its development history and clinical efficacy, the emergence of side effects, and evolving patterns of clinical use. As discussed in this review, clinical practice has adjusted usage and dosages to mitigate adverse effects, yet the need for safer delivery mechanisms persists. rhBMP-2 remains a potent osteoinductive agent with comparable fusion success, as measured by radiographic fusion and good clinical outcomes, to autologous grafts but poses unique risks. This review sets out how further research is essential to optimise the delivery of rhBMP-2 to reduce side effects. Enhanced understanding and innovation of spatio-temporal presentation relative to endogenous BMP could significantly improve patient outcomes in spinal fusion surgeries. The review contributes to the growing body of literature on the use of rhBMP-2 in spine surgery and discusses changing patterns of clinical use over time.

## Introduction and background

Spinal fusion is a well-established procedure in the United States for the treatment of degenerative spinal diseases. Previous research shows that this operative treatment has been growing in recent decades. Based on the projections of rapidly ageing populations in other industrialised countries, there is forecast to be enormous growth in demand for these procedures and associated increased healthcare pressure and cost [1].

Multimorbidity, the coexistence of two or more chronic conditions, is common among older adults, with its prevalence increasing with age [2]. The Charlson Comorbidity Index (CCI), first reported in 1987, is useful for evaluating medical comorbidities [3], and patients with higher CCI scores have been found to have poorer postoperative spinal fusion outcomes [4]. Wu et al. have reported a significant increase over time, from 2004 to 2021, in the comorbidity burden of patients undergoing lumbar fusions [5].

Given the increasing population age and rising prevalence of patients with suboptimal bone mineral density [6] and increasing comorbidities [5], bone grafts that can tilt the scales in favour of early solid fusion are particularly important. One such bone graft, Infuse™ bone graft, a combination product containing recombinant human bone morphogenetic protein-2 (rhBMP-2) delivered on an absorbable collagen sponge (ACS), was launched in 2002 (marketed as InductOs™ in Europe). Despite significant controversy, it remains the market-leading bone graft substitute globally. However, since Infuse™ is the only rhBMP-2 product approved by the Food and Drug Administration (FDA), our clinical understanding of rhBMP-2 has been largely influenced by its delivery via ACS rather than by studies focusing solely on the molecule's properties. This paper discusses the history of the molecule, its approval as a combination product, the emergence of side effects, and the impact of those side effects on clinical usage patterns.

Methods

A narrative review was conducted to compile and summarise the literature on rhBMP-2 in spinal fusion surgery over time, from the first synthesis of the recombinant version of the protein, its preclinical development, and the evolving pattern of clinical usage since launch.

Articles were selected according to the following approach and considerations. To maintain focus on the most clinically relevant applications of rhBMP-2, only studies or technical reviews evaluating rhBMP-2 in spinal fusion were included, whilst studies of other recombinant forms of bone morphogenetic protein (BMP) (e.g. rhBMP-7) were not included. Furthermore, only studies directly related to endogenous BMP-2 and the FDA-approved version of rhBMP-2 (Infuse™) were included. Inclusion was not restricted by the operative approach, but preference was given to studies using the cleared Infuse™ anterior lumbar interbody fusion (ALIF) indication. Studies involving non-spinal surgery (e.g. trauma and dental) were excluded. 

A search of published reports was performed using PubMed, and information in the public domain specifically related to the Infuse/InductOs product was also considered. The product-specific information sources included the FDA Summary of Safety and Effectiveness, European Medicines Agency (EMA) technical reviews, Infuse™ Instructions for Use, and Medtronic promotional materials. To mitigate bias from using product-specific documents, only product characteristic data was used (e.g. pack size, dose concentration, soak time). PubMed searches identified articles published from January 1980 to January 2024. To maximise the sensitivity of the search strategy, the terms "bone morphogenetic proteins", "BMP", "rhBMP-2", "spinal fusion", "ALIF", "spinal fusion", "Infuse Bone Graft", "InductOs", "side effects", "signalling", "mechanism of action", "clinical outcomes", "patterns of usage", "trends", and "temporal" were combined variously as either keywords or Medical Subject Headings (MeSH) terms. The initial searches yielded a substantial number of articles (702), from which duplicates were removed (48). The remainder (654) were then screened for relevance to the topic based on their titles, keywords, and abstracts ("abstract review"). Full-text articles (162) were retrieved and further evaluated for inclusion in this review ("paper review"). During the review process, certain references within the papers under review were pursued where the line of argument being proposed warranted it. This led to the inclusion of papers and articles not returned in the initial PubMed search. 

## Review

What is BMP-2?

BMP-2 is a member of the transforming growth factor-beta (TGF-β) superfamily [7], critically involved in osteogenesis. BMP-2 binds to type I and type II serine/threonine kinase receptors on the surface of various cell types, including mesenchymal stem cells (MSCs), initiating a signalling cascade predominantly via the suppressor of mothers against decapentaplegic (SMAD) pathway [8]. This cascade culminates in the transcription of osteogenic genes, driving the differentiation of MSCs into osteoblasts, the bone-forming cells [9,10]. BMP-2 effectively promotes the formation and mineralisation of new bone tissue, playing an indispensable role in embryonic skeletal development and postnatal bone repair and regeneration [11].

Spatial and temporal signalling in bone repair

BMPs are highly conserved among mammals, as evidenced by their functional interchangeability across species, conserved regulatory regions, and essential roles in various developmental processes [12]. This conservation underscores the fundamental importance of BMP signalling pathways in vertebrate biology. 

A key feature of endogenous BMP-2 is that it is a highly sequestrated protein in the human body. It is stored as a precursor protein, called pro-peptide (proBMP-2), which includes a signal peptide, a prodomain, and the mature BMP-2 protein. Once cleaved, the active form of BMP-2 is released and can bind to its receptor to promote signalling [7]. Controlling the availability of BMP signals at the cell surface is crucial for preventing dysregulated cellular communication. The most investigated control mechanisms include the involvement of coreceptors at the cell surface that assist in growth factor presentation and the expression of diffusible antagonists, which complex the active growth factor to prevent it from cell surface receptor engagement [13]. These pericellular mechanisms of limiting BMP action are complemented by the multifaceted function of an intricate tissue-specific cellular microenvironment, which controls the bioavailability of BMPs [13]. The bioavailability of BMP-2 is further regulated by antagonists (e.g. noggin, chordin, and gremlin) and interactions with the extracellular matrix (ECM) in a spatio-temporal controlled manner to maintain bone homeostasis [14]. Mature BMP-2 is a homodimer with one heparin binding site in each of the N-terminal sequences that can interact with ECM components, providing a protein gradient by restricting the diffusion of free BMP-2 and thereby controlling signal regulation [15,16].

In bone repair models, there is a marked delay in peak BMP-2 expression which appears to correspond with the species' bone formation rate. As set out in the table below, small mammals reach their peak in BMP-2 expression within three days [17,18], whereas rabbits have a slower time to peak expression [19], which is slower again in humans. Hara et al. [20] reported that patients in the normal healing group in long bone fractures showed a peak of BMP-2 at one week, which declined to the index event level by weeks 3 and 4 before increasing slightly again in weeks 6-8. In a Chinese language paper with an English language abstract but without DOI [21], the authors report that in patients with normal healing, the BMP-2 increased slowly from the first to the second week. The second week represented the peak, and the levels had dropped by the eighth week. These species-dependent times to peak BMP-2 expression are summarised in Table 1 below.

**Table 1:** Time to peak BMP-2 expression in fracture and bone healing studies

Species	Time to peak BMP-2 expression following injury	Source
Mouse	1 day	Cho et al., 2002 [17]
Rat	3 days	Yaoita et al., 2000 [18]
Rabbit	7 days	Si et al., 1997 [19]
Human	7-14 days	Hara et al., 2017 [20]; Xuman et al., 2007 [21]

The temporal delay in BMP-2 peak expression during human bone healing underscores the critical need for precise control over the fine-tuned cascade. Therefore, we might anticipate that the ideal exogenous delivery of rhBMP-2 with a carrier in patients should be highly controlled, with the molecule presentation rate mimicking the natural human BMP-2 expression peak. Small deviations from the spatio-temporal controlled pattern of BMP-2 delivery may contribute to the side effects observed in patients, as discussed in this paper. 

History of rhBMP-2

Dr. Marshall Urist pioneered the concept of natural substances in bone that are responsible for regeneration and bone repair. In his original 1965 publication, Dr. Urist showed that a demineralised bone matrix (DBM) prepared from human cadaveric bone and processed to remove minerals could induce new bone formation when implanted at non-bony sites in animal models [22]. He later called these substances BMPs [23].

The clinical use of BMP was initially limited since the only source at the time was via their isolation from donor bone, and to obtain several grams (g) of BMPs, several kilograms (kg) of bones are needed [24]. However, the advances in molecular biology in the 1980s and early 1990s allowed the sequencing of BMPs, and the first version of rhBMP-2 was cloned in 1988 by a team led by John Wozney [25]. This milestone resulted in rhBMP-2 production on a significant scale, allowing for comprehensive testing and translation from a basic scientific discovery to a widely used therapeutic agent in regenerative medicine.

From the early development of rhBMP-2, it was noted that the local retention and sustained release of the molecule led to enhanced bone formation at an equivalent dose [26]. As part of the product's development, Medtronic Sofamor Danek (Memphis, Tennessee, United States) evaluated a significant number of carriers for the surface attachment and delivery of the rhBMP-2. These included the following material types, hydroxyapatite, beta-tricalcium phosphate (β-TCP), biphasic calcium phosphate, calcium sulphate, silicate glass, allograft, and DBM, before the ACS was selected based on its relative performance. In vivo, studies using radioactively labelled rhBMP-2 have shown that approximately 70-75% of the rhBMP-2 is retained on the implanted ACS after three hours and that approximately 5% of the total dose of rhBMP-2 is retained on the implanted ACS by day 14. The reported mean residence time of rhBMP-2 when delivered from ACS is between 4.6 and 5.6 days [27]. No radioactively labelled rhBMP-2 was detectable by three weeks following implantation [28,29]. This short localisation period is in apparent discord with the data shown above in Table 1. There is a rapid early availability of the molecule from the ACS at a time point when endogenous BMP-2 expression would be low during normal bone healing. Conversely, there is little rhBMP-2 retained by the ACS at two weeks, which is around the time when endogenous BMP-2 expression in humans reaches a natural peak. 

Moreover, there is an important distinction to be drawn between the localisation of rhBMP-2 and its temporal bioavailability. The bioavailability of the rhBMP-2 refers to its ability to bind to the surface of cells and initiate a signalling cascade. Previous studies have not addressed the bioavailability of the rhBMP-2 delivered on ACS, only its localisation or retention period. The ACS provides a three-dimensional scaffold for the migration of stem cells into the delivery vehicle. It is unclear if rhBMP-2 absorbed into or entangled within the collagen fibres of the ACS would still have its receptor binding sites presenting at all times. If the rhBMP-2 is always bioavailable, irrespective of being localised, we can anticipate this would introduce significant differences in the initial temporal signalling between rhBMP-2 delivered on ACS and the sequestrated, progressive release of endogenous BMP-2 in human bone healing. Our understanding of the impact of bioavailability would be greatly advanced with more research in this area. 

The early clinical adoption years

In 2002, the US FDA approved a combination product (Infuse™ bone graft) containing rhBMP-2+ACS for single-level ALIF procedures with a titanium tapered cage [29]. The Infuse™ bone graft was approved in various pack sizes, each with an rhBMP-2 dose concentration of 1.5 mg/mL. Studies have shown that 1.5 mg/mL is the maximum feasible concentration of the active ingredient dibotermin alfa (rhBMP-2), with higher concentrations leading to precipitation when added to the ACS [27]. Further supplemental approvals to expand its use were granted by July 2004. These included the Inter Fix™ threaded fusion device and Inter Fix™ RP threaded fusion device, increasing the levels of use from L4-S1 to L2-S1 and allowing the device to be used in subjects who may have retrolisthesis in conjunction with degenerative disc disease (DDD).

**Figure 1:**
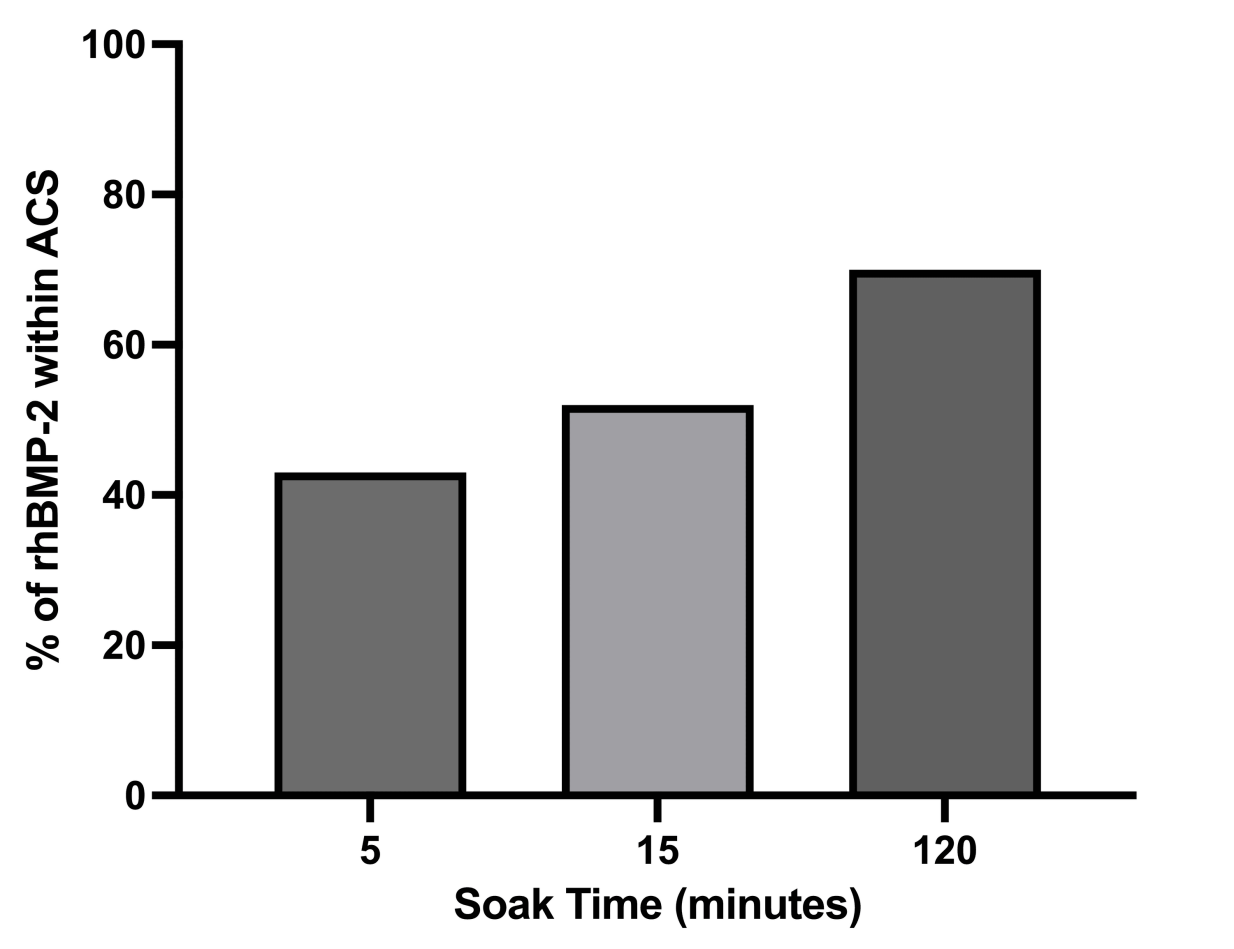
Absorption of rhBMP-2 to the ACS. Data from Infuse Bone Graft Technical Review, MLITINLTTR2 ACS: absorbable collagen sponge

As per the Instructions for Use for the Infuse™ bone graft, the lyophilised rhBMP-2 molecule is solubilised with water to produce an rhBMP-2 solution in a low-pH buffer and allowed to soak onto an ACS for a minimum of 15 minutes. Medtronic Sofamor Danek, in a promotional brochure entitled "Infuse Bone Graft+LT Cage Lumbar Tapered Fusion Device Technical Review" (brochure code MLITINLTTR2), reported that ~52% of the rhBMP-2 is absorbed onto the ACS after 15 minutes, which increases to ~70% after two hours of soaking time, as shown in Figure 1 below.

Following FDA approval, the initial clinical adoption of rhBMP-2+ACS was brisk, and it quickly became the market-leading bone graft substitute product in the United States. Several initial studies reported excellent fusion rates of the surface-attached rhBMP-2+ACS, with reduced operative time, lower blood loss, decreased length of hospital stay, and the avoidance of second-site morbidity compared to traditional autologous iliac crest bone graft (ICBG) [30-32].

Usage grew at a compound annual growth rate (CAGR) of 69% between 2002 and 2010, from less than 1% to 29% of total fusion procedures [33]. However, Ong et al. reported that at least 85% of rhBMP-2+ACS usage was off-label between 2002 and 2007 [34]. The authors report that the predominant use of rhBMP-2+ACS was in primary posterior lumbar interbody fusion (PLIF)/transforaminal lumbar interbody fusion (TLIF) (30%), followed by primary posterolateral spine fusion (PLF) (20.4%), primary ALIF (16.6%), primary cervical fusions (13.6%), and primary thoracolumbar fusions (3.9%). Of the potentially on-label ALIF procedures, it is unknown how many used the approved cages (LT-CAGE or Inter Fix fusion device, Medtronic Sofamor Danek) to ensure consistency with the labelling. 

The controversy years

From as early as 2006, studies started to emerge highlighting side effects of rhBMP-2+ACS not previously reported in earlier studies [35-38]. On July 1, 2008, the FDA issued a public health notification regarding rhBMP-2+ACS in cervical spine fusion. The FDA warned of life-threatening complications associated with rhBMP2+ACS when used off-label in the cervical spine. In 2011, Carragee et al. published a critical review of the use of rhBMP-2+ACS in spinal surgery, reporting a complication rate range of 20-70% [39]. In June 2011, the Wall Street Journal reported that the federal government was investigating Medtronic for off-label use of the Infuse™ product [40]. On June 21, 2011, the US Senate Finance Committee staff initiated an inquiry into whether Medtronic improperly influenced peer-reviewed studies of the Infuse™ bone graft and found that Medtronic was heavily involved in drafting, editing, and shaping the content of medical journal articles authored by its physician consultants [41].

Following these events, two systematic reviews and meta-analyses conducted by the Yale University Open Data Access Project (YODA) were published to comprehensively articulate the evidence of the safety of the rhBMP-2+ACS product, amid increasing uncertainty [42,43]. These analyses revealed that there was no statistically significant difference between rhBMP-2+ACS and the gold standard of autograft ICBG in terms of rates of overall success and fusion at 24 months after surgery. Furthermore, there were comparable rates of retrograde ejaculation and neurological complications after rhBMP-2+ACS to those with ICBG use in ALIF and PLF. These reviews did, however, find that there were significantly higher rates of complications associated with rhBMP-2+ACS when used in anterior cervical surgery and higher rates of ectopic bone formation in PLF. 

The impact on clinical practice

The clinical response to these findings was constrained by the fixed 1.5 mg/mL dose concentration, irrespective of the pack size, and the bioavailability of rhBMP-2 from the supplied ACS delivery vehicle. However, there have been two notable changes in clinical practice since the emergence of side effects.

Reduction in Usage

By 2015, the use of rhBMP-2+ACS in all fusion procedures had fallen to approximately 40% of its peak usage in 2010, with the most significant decrease occurring in patients between 18 and 54 years old [33]. Beschloss et al. reported a similar decrease in use among younger patients [44]. This patient cohort usually has superior bone quality and regenerative potential relative to elderly patients; thus, these patients have a modified risk versus reward calculation. 

There has also been a notable shift in fusion types which use rhBMP-2 [45]. There appears to be an association with the FDA's warning regarding potentially dangerous anterior soft tissue swelling in the neck following anterior cervical fusions (ACF), with such operations falling by 63% from a peak of 11.1% among all ACFs in 2009 to 4.1% among all ACFs in 2015, a 2.7-fold reduction. The use of rhBMP-2+ACS in PLF also had a 2.2-fold reduction from its peak of 44% in 2010. Its use in ALIF also declined from its peak, in this case, a 1.7-fold reduction.

Guzman et al. reported reductions in both primary and revision spinal fusion surgeries using rhBMP-2+ACS between quarter 1 of 2010 and quarter 4 of 2013. The data shows a larger reduction in primary surgeries (-53%) than in revision cases (-45%) [46]. Revision case fusions are considered more challenging, and this difference in reduced usage percentages is likely a reflection of the greater clinical need in revision cases [47]. Patel et al. reported a slight decline in the usage of rhBMP-2+ACS in a self-reporting survey of 142 spine surgeons. A total of 30.39% of respondents who use rhBMP-2 reported a decrease in usage between 2017 and 2022, 21.57% reported an increase over the same period, and 48.04% reported no change in their frequency of use [48].

The COVID-19 pandemic saw a reduction in lumbar fusion rates reported in a US national deidentified database derived from a large, adjudicated claims data warehouse containing commercial and Medicare US patient data [5]. A published survey of the Lumbar Spine Research Society provides data on the nature of this reduction, with a significant decrease in elective surgeries in the early months of the pandemic, whilst emergency procedures remained largely unchanged, even when the patient's COVID-19 status was unknown [49].

Utilising a commercial database (Clarivate), which incorporates data from the Centers for Medicare and Medicaid Services (CMS), the Healthcare Cost and Utilization Project (HCUP), and the Centers for Disease Control and Prevention (CDC), the number of spinal fusion procedures was returned for International Classification of Diseases 10th revision (ICD-10), Current Procedural Terminology (CPT), and Procedure Code (PX) codes for spinal fusion. Between 2019 (pre-pandemic) and 2022 (post-pandemic), there was a change in the overall number of spinal fusion procedures performed in the United States. The number of spinal fusion procedures performed in 2020 increased by +0.6% over 2019, with procedure volumes then declining in 2021 by 0.3% due to the COVID-19 pandemic before returning to growth again in 2022, with a robust increase of 3.4% during the year.

**Figure 2:**
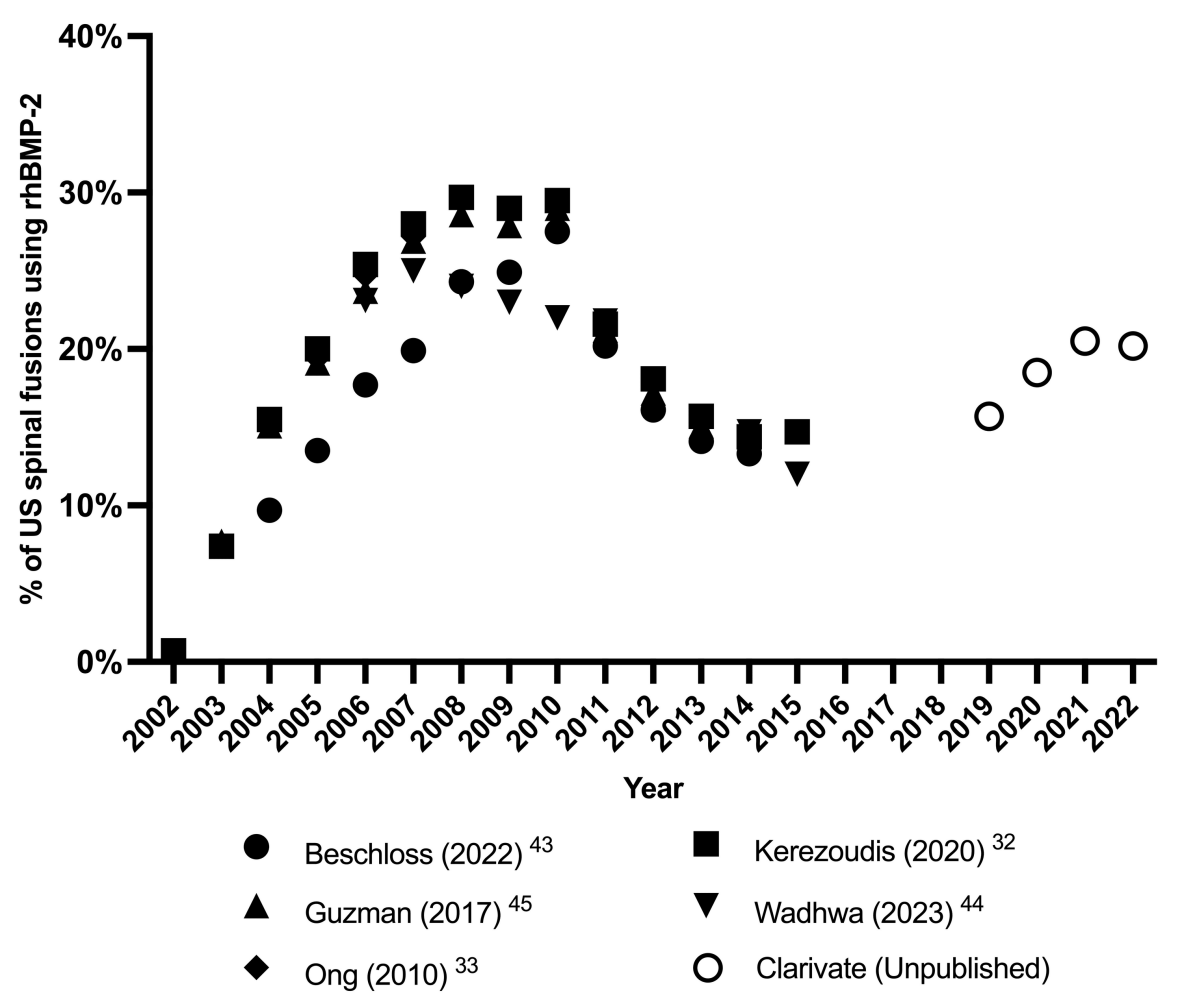
Analysis of rhBMP-2+ACS usage relative to the total spinal fusions in the United States over time ACS: absorbable collagen sponge

However, disaggregating the data shows an offset between an increase in rhBMP-2+ACS usage and declines in those procedures that did not report rhBMP-2+ACS usage. The absolute usage of rhBMP-2+ACS thus increased yearly from 2019, whereas there were falls yearly for non-rhBMP-2 procedures. This resulted in an increase from 16% of all spinal fusion procedures using rhBMP-2 in 2019 to 20% by 2022. The evolution of rhBMP-2 usage in the United States, as reported by various sources, is shown in Figure 2 below.

Reduction in Dose 

Prior to the approval of the Infuse™ bone graft, Medtronic Sofamor Danek sponsored several ALIF studies. When reviewed retrospectively and sequentially, there was a clear trend towards using higher doses per level over time, despite the successful fusion rates seen at the lower doses per level, following a "more is better" philosophy. The dose per level data from those early Infuse™ studies are set out below in Table 2. 

**Table 2:** Summary of the early ALIF clinical studies using rhBMP-2+ACS ALIF: anterior lumbar interbody fusion; ACS: absorbable collagen sponge; I*: investigational group; C**: control group

#	Approach	Start date	Reference	Study name	Sample size		Dose concentration (mg/mL)	Total dose per level (mg)
					I*	C**		
1	ALIF	1997	Boden et al., 2000 [50]	LT-Cage Pilot	11	3	1.5	3.9-7.8
2	ALIF	1998	Burkus et al., 2002 [30]	LT-Cage Pivotal (open)	142	136	1.5	4.2-8.4
3	ALIF	1998	Burkus et al., 2002 [30]	LT-Cage Pivotal (laparoscopic)	134	-	1.5	4.2-8.4
4	ALIF	1998	Burkus et al., 2002 [31]	Bone Dowel Pilot	24	22	1.5	8.1-11.7
6	ALIF	1999	Unpublished	Interfix™ Pilot	25	20	1.5	8.4-16.8
7	ALIF	2000	Burkus et al., 2005 [51]	Bone Dowel Pivotal	55	30	1.5	8.1-11.7
8	ALIF	2003	Gornet et al. 2011 [52]	Infuse™ vs Maverick™ Pivotal	172	405	1.5	4.2-12.0

Notably, with each industry-sponsored trial publication, all of the safety findings reported no adverse events associated with the device within the investigational treatment arm despite approaching 600 patients. This apparent under-reporting was met with significant criticism from Carragee et al. [39]. Reporting adverse events is a critical safety check in adopting new technologies, and its under-reporting is likely to have contributed to the use of rhBMP-2+ACS at higher doses. When developed, the Infuse™ dose was intended to be volume-dependent, with the Infuse™ volume equalling the internal volume of the cage; however, surgeons can underfill or overfill the cage, varying the delivered dose of rhBMP-2 [53].

As noted above, since BMP-2 in normal physiology is a highly sequestered protein, controlling the availability of BMP signals at the cell surface is crucial for preventing dysregulated cellular communications [13]. A combination of too high a dose and the rapid availability of rhBMP-2 from ACS would be expected to contribute to dysregulated signalling, as both are in contrast to the progressive and delayed peak BMP-2 expression in human bone healing [20].

The emergence of rhBMP-2+ACS side effects led authors, including Bae et al. [54] and Toth et al. [55], to investigate the relationship between dose and side effects in large animal preclinical studies. Both authors showed that overfilling graft sites with high doses of rhBMP-2+ACS are associated with significant short-term osteoclastic activity, leading to bone resorption [54,55].

Unable to modify the dose concentration of 1.5 mg/mL, clinicians have sought to reduce the risk of side effects by reducing the total implant volume and, therefore, the total rhBMP-2 dose, leading to a general decline in the dose per level over time. De Stefano et al. performed a retrospective study of 1,209 patients who received rhBMP-2+ACS between 2006 (the start of reports of side effects) and 2020 [56]. They showed a significant decrease in the rhBMP-2 dose used per level. The most significant decrease was in PLIF/TLIF surgical approaches, decreasing from a mean of 5.97 mg/level in 2009, with 2011 witnessing a significant decrease to 4.09 mg/level in the rhBMP-2 dose used, coinciding with the Carragee et al. paper [39], and still further to a mean of 1.35 mg/level in 2020 (p<0001). 

Bannwarth et al. [57] conducted a retrospective review of the International Spine Study Group prospective multicentre database to identify adult spinal deformity (ASD) patients treated surgically from 2008 to 2018. They found that the overall dose of rhBMP-2+ACS decreased over time. The authors speculate that multiple factors could account for this decline, including attempts to reduce cost, increased surgeon experience favouring acceptable fusion rates with lower doses, and concerns for potential associated complications [57].

McGrath et al. reported the impact of cost-awareness training on clinicians, showing a downward change in surgeon dose decisions pre- and post-cost-awareness training [58]. McGrath et al.'s findings support Bannwarth et al.'s speculation that down-dosing is, at least in part, influenced by cost.

Has the dose reduction been effective in reducing side effects?

Hofstetter et al. [59] published a meta-analysis of dose-related efficacy and morbidity of rhBMP-2 in spinal arthrodesis surgery. Their meta-analysis shows that the total rhBMP-2 dose used varied considerably between studies and spinal arthrodesis procedures, presumably because of the difference in cage sizes by approach. Their comprehensive publication included nine studies reporting rhBMP-2+ACS clinical efficacy and complications following ALIF [59]. In their meta-data analysis, they found that the use of rhBMP-2+ACS led to a statistically significant increase in fusion rates in ALIF versus control and that the fusion rates were similar among various doses of rhBMP-2+ACS analysed. When combining reports in which various rhBMP-2+ACS concentrations were used, they found a trend toward a higher rate of complications with rhBMP-2+ACS compared with procedures performed without rhBMP-2+ACS. 

They found that using a low dose (<4.2 mg) of rhBMP-2+ACS per level incurred a rate of complications of 7%, whereas using a high dose (>8.4 mg) per level was associated with a 17.7% rate of complications. The rate of complications exhibited a significant positive correlation with the dose of rhBMP-2+ACS used per level (Pearson correlation coefficient 0.98, p<0.05). Thus, within the papers analysed by Hofstetter et al., surgeons could significantly reduce, but not eliminate, a higher rate of side effects when using rhBMP-2+ACS in ALIF procedures. The most frequent rhBMP-2+ACS-related complications in the meta-analysis were retrograde ejaculation (6.7% vs 2.7% in the control group) and endplate resorption (12.5% vs 0% in the control group) [59].

## Conclusions

Clinicals and healthcare systems are forecast to face a significant increase in demand for spinal fusions. With the rapidly ageing global population, more of their patients will be elderly and have more comorbidities. rhBMP-2 has been shown to be a powerful osteoinductive agent, with equivalent fusion outcomes to the gold standard of ICBG, whilst overcoming the morbidity associated with ICBG harvesting. However, when delivered on ACS, rhBMP-2 has been reported to cause side effects not produced by endogenous BMP-2 in bone healing. Over time, surgeons have been able to modify the patient benefit-risk assessment by reducing the total dose relative to the higher doses used in the first few years of clinical adoption and reducing use in higher-risk approaches like ACF. Despite this, the risk of side effects of rhBMP-2+ACS persists. 

Whilst the retention of the rhBMP-2 delivered on ACS has been reported and discussed above, little research has investigated the temporal bioavailability or signalling availability of the rhBMP-2 delivered in this way. Given the delay in temporal signalling in human bone repair, an absence of the progressive presentation of the active form of rhBMP-2 may contribute to dysregulated cellular communication and the side effects reported with rhBMP-2+ACS. Retention, or localisation, may be a necessary, but insufficient, condition for the elimination of side effects. There is an urgent need for greater research in this area. The potential for future products to modify the bioavailability of rhBMP-2 and thus better mimic the temporally delayed peak in BMP-2 expression and signalling in higher-order animals may contribute significantly to the reduction of persistent side effects.
